# Time-course profiling of bovine alphaherpesvirus 1.1 transcriptome using multiplatform sequencing

**DOI:** 10.1038/s41598-020-77520-1

**Published:** 2020-11-24

**Authors:** Norbert Moldován, Gábor Torma, Gábor Gulyás, Ákos Hornyák, Zoltán Zádori, Victoria A. Jefferson, Zsolt Csabai, Miklós Boldogkői, Dóra Tombácz, Florencia Meyer, Zsolt Boldogkői

**Affiliations:** 1 Department of Medical Biology, Faculty of Medicine, University of Szeged, Somogyi B. u. 4., 6720 Szeged, Hungary; 2 Institute for Veterinary Medical Research, Centre for Agricultural Research, Hungária krt. 21, 1143 Budapest, Hungary; 3 Department of Biochemistry and Molecular Biology, Entomology and Plant Pathology, Mississippi State University, 408 Dorman Hall, 32 Creelman St., Box 9655, Starkville, MS 39762 USA

**Keywords:** Gene expression analysis, Gene expression profiling, Transcriptomics, RNA sequencing, Virology, Transcriptomics

## Abstract

Long-read sequencing (LRS) has become a standard approach for transcriptome analysis in recent years. Bovine alphaherpesvirus 1 (BoHV-1) is an important pathogen of cattle worldwide. This study reports the profiling of the dynamic lytic transcriptome of BoHV-1 using two long-read sequencing (LRS) techniques, the Oxford Nanopore Technologies MinION, and the LoopSeq synthetic LRS methods, using multiple library preparation protocols. In this work, we annotated viral mRNAs and non-coding transcripts, and a large number of transcript isoforms, including transcription start and end sites, as well as splice variants of BoHV-1. Our analysis demonstrated an extremely complex pattern of transcriptional overlaps.

## Introduction

Bovine alphaherpesvirus 1.1 (hereon referred to as BoHV-1) is a large (~ 136 kbp) double-stranded DNA virus that belongs to the *Alphaherpesvirinae* subfamily. BoHV-1 largely infects cattle and related ruminants. BoHV-1 is one of the viruses that triggers the disease commonly known as bovine respiratory disease (BRD), which costs the cattle industry several billion dollars annually worldwide^1^. Similarly to other alphaherpesviruses, such as herpes simplex virus type 1 (HSV-1), or pseudorabies virus (PRV), after 7–10 days of acute infection in the mucosal epithelium, BoHV-1 gains access to axons of peripheral sensory neurons, travels in retrograde fashion up the axon to establish latency in the neuronal body housed in the trigeminal ganglion (TG). The viral genome is retained in neurons throughout life^2^. With the exception of the LR-gene^3^, gene expression during latency is undetectable. A variety of stress signals can trigger BoHV-1 out of its latent state, re-establishing a productive infection at the epithelium of initial infection, promoting viral spread^2^. Although the primary site of BoHV-1 latency is sensory neurons within TG, long-term persistence and reactivation can also occur within germinal centers of pharyngeal tonsil^4^.

The viral genome has been sequenced and structurally annotated by an international effort more than 20 years ago, using viral DNA from several strains/subtypes^3–5^. To date the original genomic annotation of 69 open reading frames (ORFs)^6^ has not been revised. Several new ORFs are now recognized to exist in and around the latency-related (LR) gene^7,8^, and recently a new ORF was identified using experimental proteomic data^9^. Proteogenomic data has also revealed at least 92 unannotated peptides coded by the BoHV-1.1 genome, 21 of which are surrounded by potential ORFs identified *in* silico^9^*.* The herpesvirus genes can be classified as immediate-early (IE), early (E), early-late (L1) and late (L2) genes depending on the kinetics they are expressed throughout the viral replication cycle^10^. ORFs of the BoHV-1 genes are mainly annotated in silico, whereas the size and the possible splicing patterns of many transcripts was detected using Northern blot and S1 nuclease mapping^4,11–13^. Until now, the only genome-wide transcriptome assay focused on the detection of microRNAs of BoHV-1^14^.

Next-generation sequencing (NGS) [short-read sequencing (SRS)] technology has revolutionized transcriptome research due to its capacity to sequence a large number of nucleic acid fragments simultaneously, at a relatively low cost. In the last couple of years, third-generation sequencing (TGS) [long-read sequencing (LRS)] has become an alternative approach that is able to circumvent the limitations of SRS, including its inability to read full-length RNA molecules that are necessary, among others, for the identification of transcript isoforms and for distinguishing between overlapping transcripts. Recently, TGS has been widely applied for the transcriptome analysis of a variety of organisms^15–27^, including herpesviruses^21,28–34^. These approaches have revealed a much more complex transcriptional landscape of the examined viruses than previously thought.

In this study, we carried out a time-lapse analysis of BoHV-1 transcriptome using two LRS techniques, ONT MinION and Loop Genomics LoopSeq™ synthetic long-read sequencing on the Illumina platform.

## Results

### Time-lapse transcriptome analysis of BoHV-1 using long-read sequencing

In this work, we carried out a time-course analysis of the BoHV-1 transcriptome. In order to capture the most complete transcriptome dataset, we applied various experimental conditions (different cell types for virus propagation, different multiplicities of infection, various library preparation methods and sequencing platforms). Transcriptome analysis was performed using two LRS technologies: ONT nanopore sequencing and LoopSeq synthetic long-read sequencing on Illumina MiSeq platform. We applied cDNA and direct RNA sequencing (dRNA-Seq), as well as oligo(dT) and random oligonucleotide-primed reverse transcription for the ONT platform. We also used an amplified cDNA-based technique in pooled samples and a non-amplified cDNA-based technique in time-variant samples. For transcript annotation, mapped reads were analyzed using the LoRTIA software suite developed in our laboratory.

In previous publications, we^20,27^ and others^35^ have reported that the dRNA-Seq method is not suitable for capturing complete transcripts, since short (15–30 bps) sequences from the 5′ region and in many cases also the poly(A)-tails were missing from the reads. The lack of 5′-ends is the result of the release of the RNA strand from the ratcheting protein leading to the rapid transition of RNAs through the pore. On the other hand, polyA-tails are probably missing due to the presence of a DNA adapter ligated to the 3′-end during library preparation. This adapter muddles the signal near the 3′-ends of the RNA molecule, resulting in erroneous base calling.

Another drawback of native RNA sequencing is its relatively low throughput compared to cDNA sequencing. On the other hand, dRNA-Seq is free of artefacts produced by RT, second strand synthesis, and PCR. We used random oligonucleotide-primed cDNA synthesis for transcriptional start site (TSS) and splice site validation. Three biological replicates were prepared for each time-point in the dcDNA sequencing experiment, which showed high reproducibility (Supplementary Fig. S1).

Altogether, 29 reactions were run using 5 different techniques for providing independent reads. The RT, the second strand synthesis and the PCR often result in nonspecific binding of oligod(T) or PCR primers and in template switching. It has been demonstrated that oligo(dT) primers can occasionally hybridize to A-rich regions of the mRNA or to the first cDNA strand and thereby producing false transcription end sites (TESs) or truncated 5′-ends^36^. Such products were eliminated from further analysis by the LoRTIA software.

The LoRTIA toolkit detected a total of 2870 putative transcription start sites (TSSs), 475 putative TESs, and 645 putative introns in the MinION sequencing data (Supplementary Table S1). TSS and TES were accepted if the LoRTIA pipeline detected them in at least three independent samples. Applying this filter resulted in 823 TSSs and 135 TESs, which were used for transcript annotation. Altogether, 25 introns were identified using our filtering criteria, among which 23 carried canonical GT/AG and 2 GC/AG splice junction sequences (Table 1).

**Table 1:** Introns annotated in the BoHV-1 transcriptome.

Start	End	Length	Read count	Strand	Junction consensus
22,945	23,500	555	1941	−	GT/AG
76,461	78,040	1579	44	−	GT/AG
76,461	79,835	3374	2643	−	GT/AG
102,951	108,437	5486	141	−	GT/AG
102,954	108,437	5483	72	−	GT/AG
107,968	108,437	469	427	−	GT/AG
111,899	112,011	112	18,853	+	GT/AG
112,077	112,210	133	11,660	+	GT/AG
112,077	112,408	331	4	+	GT/AG
112,077	112,443	366	5	+	GT/AG
112,077	112,453	376	9	+	GT/AG
112,095	112,210	115	81	+	GC/AG
112,284	112,502	218	22,488	+	GT/AG
112,284	112,524	240	9	+	GT/AG
112,571	112,865	294	24,661	+	GT/AG
125,197	125,491	294	23,449	−	GT/AG
125,538	125,778	240	11	−	GT/AG
125,560	125,778	218	21,948	−	GT/AG
125,619	125,985	366	5	−	GT/AG
125,629	125,985	356	4	−	GT/AG
125,634	125,985	351	6	−	GT/AG
125,852	125,967	115	74	−	GC/AG
125,852	125,985	133	11,737	−	GT/AG
126,051	126,163	112	18,282	−	GT/AG
129,625	130,094	469	429	+	GT/AG

The read counts are the raw total counts resulting from the LoRTIA analysis. Nucleotide positions are given based on the genome with accession number JX898220.1.

The selected TSSs, TESs and introns, used for transcript annotation, yielded a total of 1381 putative transcripts. We excluded putative transcripts detected in less than three samples, which resulted in a total of 1025 transcripts (Table 2).

**Table 2:** Transcript isoform annotated in BoHV-1.

	Count
**Non-coding**	
5′ truncated (-TR)	43
Non-coding	44
Antisense (-AS)	10
**Coding**	
Novel ORF	1
Possibly protein-coding	152
Long 5′-UTR (-L)	451
Short 5′-UTR (-S)	132
Alternative termination (-AT)	31
Monocistronic	44
Bicistronic	21
Tricistronic	15
Polycistronic	8
Complex (-C)	76
Spliced	19
Non-spliced	2

Our investigations revealed that the entire BoHV-1 genome is transcriptionally active (including the genomic junction) (Supplementary figure S2) with the exception of a 336-nt region between the TESs of CIRC and UL54, as well as a 1638-nt genomic segment between the TSS of bICP4 and the TES of ORIS-RNA1.

### Novel putative protein-coding genes: embedded genes

In principle, all of the transcripts should be considered as new because conventional techniques were unable to detect full-length transcripts and therefore match identified TSS and TES sequences. However, we only consider the transcripts containing 5′-truncated in-frame ORFs as novel putative mRNAs. These transcripts are produced by promoters located within larger canonical ORFs, or theoretically, in those cases where no promoters were identified by polymerase slippage, a mechanism which—among herpesviruses—has only been described in KSHV^37^. LRS techniques have proven to be very useful for the detection of the hidden complexity in embedded ORF-containing transcripts^21,38^. In this work, we identified 152 5′-truncated transcripts with in-frame ORFs (data not shown).

### Long non-coding RNAs

#### Antisense ncRNAs (asRNAs)

**Figure 1:**
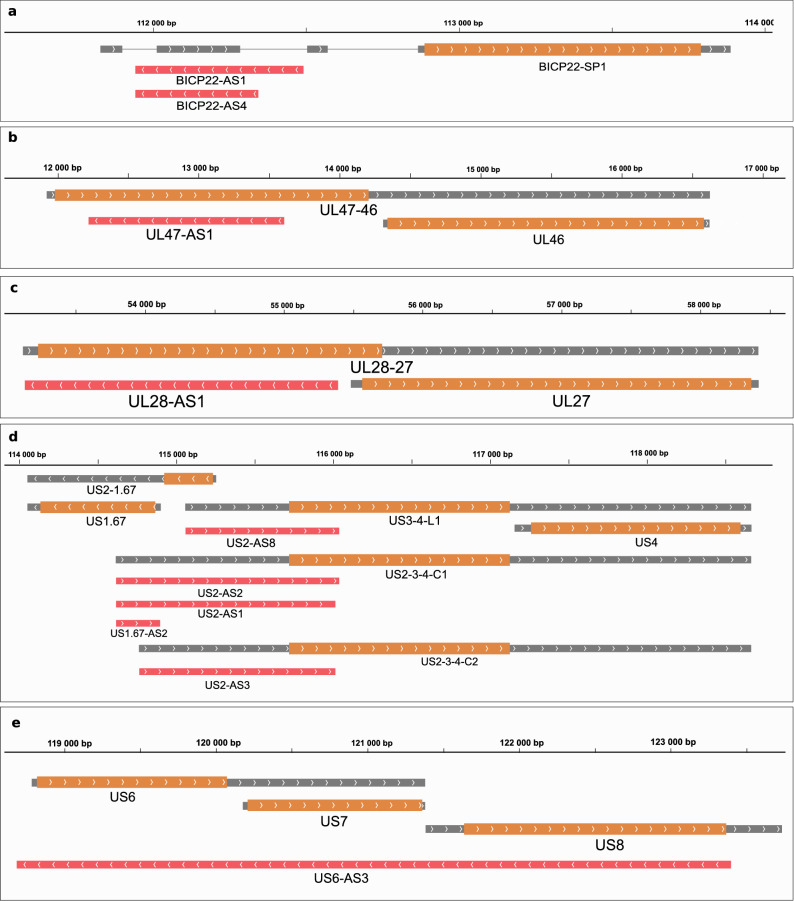
Antisense transcripts of the BoHV-1. Grey rectangles represent mRNAs. Wider overlapping orange rectangles are ORFs. Lines between rectangles represent introns. The orientation of transcripts is represented by arrow heads. Red rectangles represent antisense transcripts. (**a**) Two co-terminal antisense transcripts were detected overlapping the 5′ UTR of bICP22-SP1. (**b**) An antisense transcript overlaps the ORF of ul47. (**c**) One antisense transcript was detected overlapping the ul28 ORF. (**d**) Several antisense transcripts were detected overlapping us1.67 and us2. These start at the same TSS as the transcript isoforms of the adjacently located genes us3 and us4. (**e**) A very long antisense transcript overlaps the us6, us7 and us8 genes.

This study detected ten asRNAs each with distinct promoters. bICP22-AS1 and bICP22-AS4 are co-terminal with each-other and overlap the 5′-UTR region of bICP22 (Fig. 1a). UL28-AS1, and UL47-AS1 overlap the ORFs of the UL28 and the UL47 genes, respectively (Fig. 1b,c). Additionally, a high variety of asRNAs are transcribed from the Unique Short (US) region, resulting from premature termination of longer transcripts. US2-AS1, US2-AS2 and US1.67-AS2 are starting at the same TSS and are alternatively truncating isoforms of the very long US2-3-4-C1 complex transcript. In the same way, US2-AS3 is a 3′-truncated isoform of US2-3-4-C2, whereas US2-AS8 is a 3′-truncated isoform of US3-4-L1 (Fig. 1d). US6-AS3 overlaps three ORFs in antisense orientation (Fig. 1e). An 1128 nt-long ORF is located on this transcript but its 376 amino acid long putative protein product does not show similarity to any other proteins in the non-redundant protein databases of NCBI.

**Figure 2:**
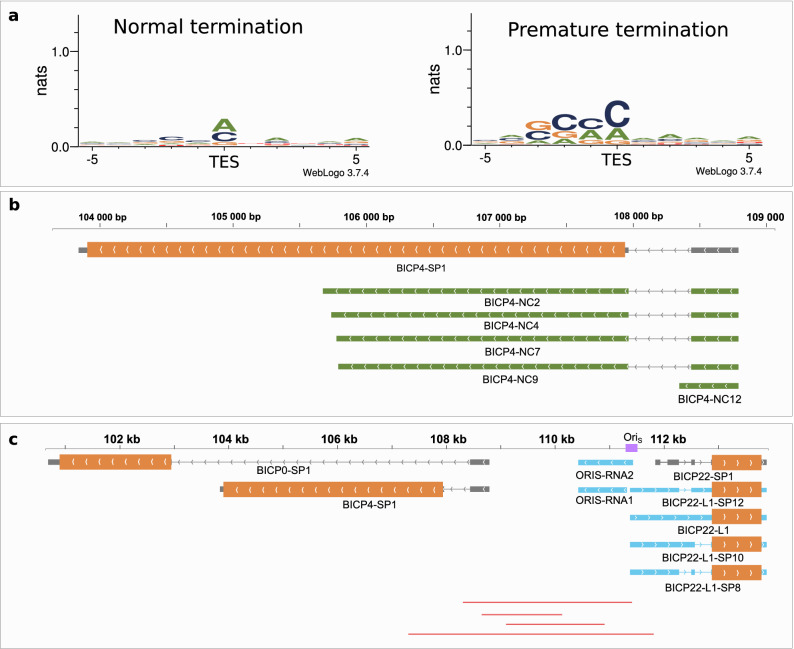
Non-coding transcripts and raRNAs of BoHV-1. (**a**) The ± 5 bp sequence surroundings of the most frequent TESs and the TESs of 3′ truncated transcripts. TESs of prematurely terminating transcripts are characterized by GC-richness immediately upstream of the cleavage site. The unit of entropy is based on natural logarithm and is expressed in natural units of information (nats). (**b**) Grey rectangles represent coding transcripts. The wider overlapping orange rectangle is the ORF. Lines between the rectangles represent introns. The orientation of transcripts is represented by arrow heads. Green rectangles represent 3′ truncated transcripts. Five 3′ truncated transcripts were detected overlapping the bICP4 gene. (**c**) Grey rectangles represent transcripts located close to the Ori region. The wider overlapping orange rectangles are ORFs. Lines between the rectangles represent introns. The orientation of the transcript is represented by arrow heads. Light blue rectangles represent transcripts overlapping the Ori_S_. Two non-coding RNAs and four 5′-UTR isoforms of bICP22 were detected overlapping the Ori. Red lines represent individual reads present in the intergenic region of bICP4-bICP22.

*3′-truncated ncRNAs (3tncRNA)* are the result of premature transcription termination that lead to the lack of stop codons on the mRNA. We annotated 3′-truncated long non-coding RNAs (lncRNAs) in 13 genes, totaling 44 transcript isoforms. These RNA molecules lack canonical polyadenylation signal (PAS) and the sequence directly upstream of their termination is GCA-rich, which is not the case for the main transcripts (Fig. 2a). The bICP4 gene encodes five 3′-truncated RNAs (Fig. 2b), four of which terminate in GA-rich regions. These loci may act as transcriptional pause sites, that promote termination, cleavage and polyadenylation^39^. The UL27 gene produces the highest variation of 3tncRNAs with seven truncated isoforms. Additionally, four of the six 3tncRNAs transcribed from bICP22 are spliced using the same splice sites as the main gene. Non-coding RNAs can be first detected in the second hour of the infection, and they continue to be expressed throughout the viral lifecycle (Supplementary figure S3).

The Coding Potential Assessment Tool (CPAT)^40^ suggests that 61 of the lncRNAs have ORFs with highly similar linguistic features to vertebrate coding sequences, 46 of them having a coding probability higher than 80% (Supplementary table S3).

### Replication-origin-associated transcripts

Replication of BoHV-1 starts between 2 to 4 h p.i.^41^. The viral genome contains a replication origin (OriS) in both repeat regions, but lacks OriL, which is present in its close relative PRV. Replication-associated (ra)RNAs overlap the OriS sequences. We identified six raRNAs: ORIS-RNA1 and ORIS-RNA2, and four 5′-UTR isoforms of bICP22 (bICP22-L1, bICP22-L1-SP10, bICP22-L1-SP12, bICP22-L1-SP8) (Fig. 2c). The raRNAs were present in low abundance and only at late time points of infection (8 h and 12 h).

### Transcript ends, length isoforms and promoters

#### Transcript start sites and promoters

**Figure 3:**
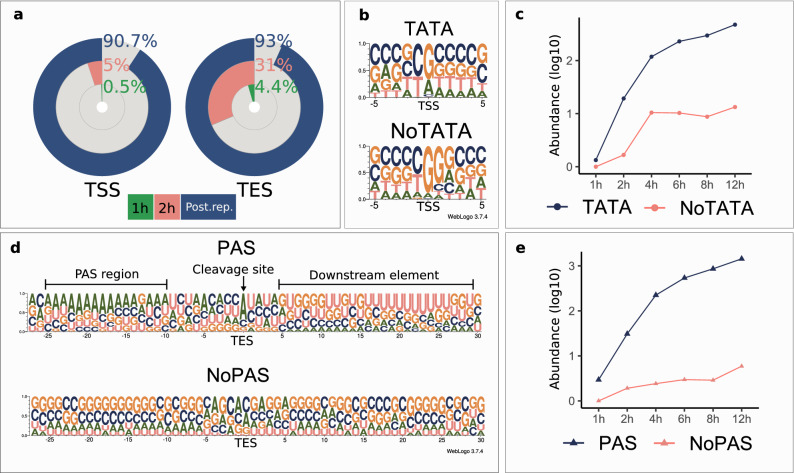
Differential usage of promoters, initiator sites and cleavage sites of the virus. (**a**) The proportion of detected TSSs and TESs at 1 h and 2 h p.i. and at postreplicative stages of the infection. (**b**) The probability of nucleotides within a ± 5 bp interval of the TSSs with or without TATA box promoters. TSSs with an upstream TATA box show a high probability of G/A being located in the TSS position, while the nucleotide in the − 1 position being either C or T. TSSs without a TATA box on the other hand are G-rich in the TSS position and the following (+ 1) nucleotide. (**c**) The cumulative log_10_ abundance of TSSs with or without a TATA box during the infection. The abundance of TSSs with and without a TATA box increase in the same manner in the IE and E phase of the infection. Following the 4 h p.i. time point, the abundance of TATA-less TSSs remains constant whereas the abundance of those with a TATA box is increasing. (**d**) The probability of nucleotides occurring in the vicinity of the TESs of transcripts with or without a PAS. TESs with a PAS have a canonical cleavage and downstream element (DE) sequence, while TESs without a PAS lack both the consensus cleavage and DE sequence motifs. (**e**) The cumulative log10 abundance of TESs with or without a PAS during the infection. The abundance of TESs with a PAS increases during the entire period of the infection, while the abundance of TESs lacking a PAS shows a steadier increase, with a pronounced steep rise between 8 and 12 h p.i.

**Figure 4:**
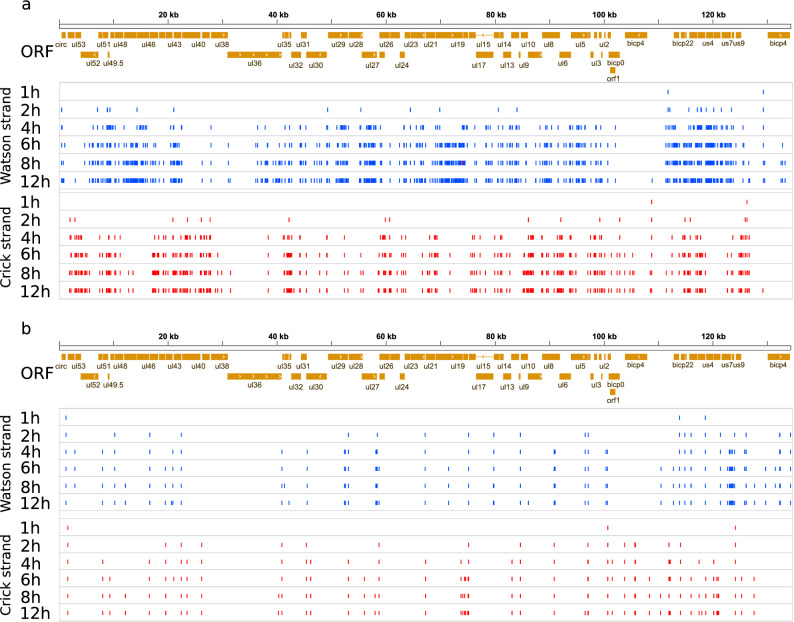
Genome-wide kinetics of (**a**) the TSSs and (**b**) TESs of BoHV-1. Blue dashes represent (**a**) TSSs or (**b**) TESs on the forward strand, whereas red dashes represent (**a**) TSSs or (**b**) TESs on the reverse strand. Orange rectangles illustrate the ORFs. TSSs and TESs were detected using the LoRTIA software suite.

In this work, we detected 823 TSSs using the criterion for sequencing reads to be present in at least three independent samples. Four of the TSSs were present in the first hour of the infection. This number increased ten times at the second hour, while 90.7% of the TSSs were only detected following the start of the viral replication (Fig. 3a). Promoter analysis disclosed 91 TATA consensus sequences at an average distance of 30.6 nt (σ = 2.09) upstream from TSS positions and fifteen CAAT consensus sequences at an average distance of 105.8 nt (σ = 17.53) (Supplementary Table S2). The eukaryotic initiator sequence region is poorly conserved in this virus (Py A N U/A)^42^. Our analysis revealed a similar consensus but with an enrichment in Gs in the + 1 position for TSSs with a TATA-box. TSSs lacking a TATA-box showed an even higher enrichment of Gs in the + 1 position followed by a predominance of Gs in the + 2 position and an ambiguous + 3 position (Fig. 3b). This G enrichment has also been described in the initiator element (INR) of HSV-1′s VP5 promoter^38,43^. TSSs with a canonical promoter sequence are more abundant at every time point in the infection, than those lacking it (Fig. 3c). We identified a putative TSS for RNA1.5 overlapping the genomic junction^44^, which was located at 129,272 nts, and it was found to be co-terminal with the more abundant CIRC RNA. We also detected splice junctions of this transcript located at positions 134,896 and 473. RNA1.5 appears to be a low-abundant CIRC variant with a longer 5′-UTR and an intron. TSS isoforms were detected starting on the second hour of infection and their expression continued throughout the entire course of infection making up 58% of all transcripts in the late phase (Supplementary figure S3). The TSS distribution alongside the BoHV-1 genome is illustrated in Fig. 4a.

#### Transcript end sites

RNA termination is more precisely regulated than the initiation. This results in a lower TES diversity. Using the above criterion for a LoRTIA transcript, we identified 135 TESs. Altogether six TESs were detected in the first hour of the infection, 31% in the second hour, whereas 90% of the TESs could be detected in the late phase of the infection. Our analysis revealed that 48 out of the 135 identified TESs have PAS consensus sequences at an average of 24.5 nt (σ = 5.98) upstream of their TESs (Supplementary Table S2). TESs with PASs have canonical sequence surroundings consistent with the eukaryotic RNA cleavage regions with an orthodox A/C cleavage site and a U/G-rich downstream element, whereas TESs without PAS did not show any consensus sequence motifs at any of the key regions (Fig. 3d) surrounding the cleavage site. TESs with canonical regulatory sequences are more abundant at every time point of the infection, than those of lacking these sequences (Fig. 3e). We identified 31 transcripts that terminated in alternative loci compared to their most abundant isoform. All TES isoforms were detected in the late phase of the infection (Supplementary figure S3). The TES distribution alongside the BoHV-1 genome is illustrated in Fig. 4b.

#### Spliced transcripts and splice isoforms

**Figure 5:**
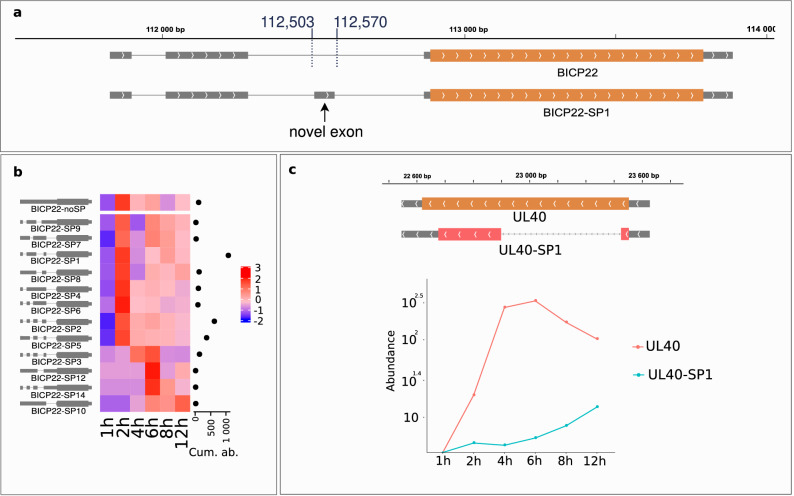
Novel splice variants of BoHV-1. Grey rectangles represent coding transcripts. The wider overlapping orange rectangles are ORFs. Lines between the rectangles represent introns. The orientation of the transcript is represented by arrow heads. (**a**) A novel 67 nt long exon was detected overlapping the second intron of the previously detected bICP22 isoform. The isoform lacking this exon was detected in very low abundance. (**b**) The change of the expression of specific bICP22 splice isoforms. The scale of the heat map represents Z-scores. The cumulative abundance of each splice isoform is represented by the horizontal dot plot on the right of the heat map. Most of the isoforms show a similar early expression pattern as the most abundant transcript, whereas bICP22-SP3, bICP22-SP10, bICP22-SP12, bICP22-SP14 are expressed more during the later phases of the infection. (**c**) A novel spliced isoform of UL40 was detected. Splicing causes a frame shift in the *ul40* gene’s ORF (red rectangle), resulting in a 104 aa-long product. The log_10_ abundance of the non-spliced and spliced isoforms is shown on the right. UL40 is characterized by an increased early gene expression and decreasing abundance starting from 6 h p.i. Its spliced isoform on the contrary has a low expression in the early phase of the infection, which starts to increase at 4 h p.i.

Compared to gammaherpesviruses, alphaherpesviruses have much fewer spliced transcripts. This is indeed true for high-abundance splice isoforms. However, as in HSV-1^38^, we also detected a number of low-abundance spliced RNA molecules in BoHV-1. In this analysis, we identified 25 introns (23 with GT/AG and 2 with GC/AG) by dRNA-Seq. All of these introns were also detected using cDNA-Seq techniques. One-third of all introns were detected in the first, 60.7% in the second hour of the infection, whereas in the late phase 82%. We identified all of the previously annotated six introns. However, one of the bICP22 introns spanning from 112,284 to 112,865^13^ was present in only two samples represented by merely 4 reads. We detected two novel abundant introns in this region, the first with the same donor and the second with the same acceptor positions as the previously annotated splice sites, but with a 67 nt-long exon between the two (Fig. 5a). We found a total of 23 novel splice variants with every intron being located in the 5′-UTR region of the transcript. We also located a non-spliced version of the bICP22. Most of the splice isoforms of bICP22 are most abundant at 2 h p.i. with the exceptions of bICP22-SP3, bICP22-SP12, bICP22-SP14 and bICP22-SP10, which peak at 6 h p.i. (Fig. 5b). A non-spliced version of UL15 starting in the same TSS and co-terminal with its spliced variant was also present in our samples, although in a lower abundance than its previously annotated spliced variant. In addition, we also detected an alternatively spliced UL15 transcript with the previously annotated intron overlapping its splice donor position at 78,040. This novel intron having a length of 3579 nts is preceded by a short exon with a start codon. The resulting 128-amino-acid-long putative protein showed no similarity with any of the proteins in the NCBI non-redundant protein database (data not shown). Finally, deleted segments found in cDNA that were absent in dRNA-seq data were only considered as *putative introns* if they were present in at least 3 independent samples. We found a total of 28 putative introns, 23 with GT/AG and 5 with GC/AG consensus (Supplementary Table S1).

**Figure 6:**
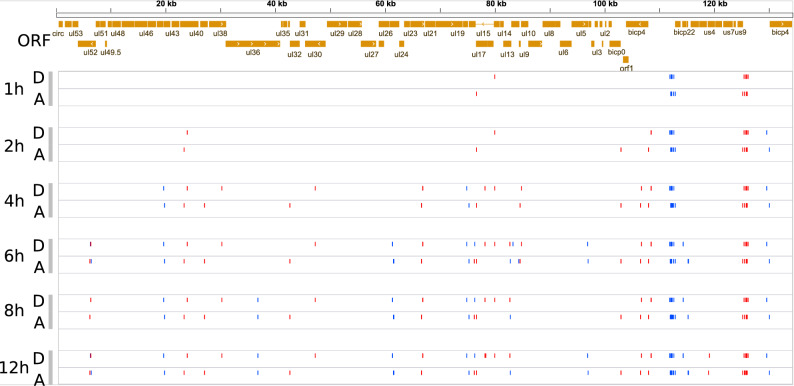
Genome-wide kinetics of splice junctions of the BoHV-1. Blue dashes represent splice junctions on the forward strand, whereas red dashes represent splice junctions on the reverse strand. Sites are plotted in two rows per sample, ‘D’ represents the donor, and ‘A’ represents the splice acceptor sites. Orange rectangles illustrate the ORFs. Splice junctions were detected using the LoRTIA software suite.

We detected a novel splice variant in a UL40 transcript. The intron spanning from 22,945 to 23,500 produces a frame shift mutation. The predicted 104 aa-long product of the UL40-SP1 showed no significant similarity to any other proteins in the NCBI non-redundant database. This intron is also present in several TSS-variants of the UL40. We observed an increased abundance of the non-spliced isoform until 6 h p.i., followed by a decrease, whereas the abundance of the spliced isoforms started to increase at 4 h p.i. and continued increasing until 12 h p.i. (Fig. 5c). Spliced isoforms could be detected in every phase of the infection. Two out of three genes detected at the first hour p.i. are spliced, however the fraction of spliced isoforms declines with the progression of the infection. The distribution of splice junctions alongside the BoHV-1 genome is illustrated in Fig. 6.

### Multigenic transcripts

#### Polycistronic transcripts

Herpesvirus genes are organized into tandem gene clusters sharing common transcription termination sequences. It is a general wisdom that in eukaryotes only the most upstream ORFs are translated from polygenic transcripts^45^. In a few examples however, translation from downstream genes of polygenic transcripts has been described in both eukaryotes^46^ and their viruses^47^. For example, it has been reported that an upstream ORF (uORF) helps the translation of the downstream ORF36 of Kaposi’s sarcoma-associated herpesvirus (KSHV) by a termination–reinitiation mechanism^48^. We detected a high number of polygenic transcripts using the stringent criteria, including 21 bicistronic, 13 tricistronic, 3 tetracistronic and 4 pentacistronic isoforms (data not shown). To identify the longest transcript isoforms in our samples, we screened the sequencing reads and annotated two more tricistronic and an additional tetracistronic transcript. These were represented by a single read, thus we deem their start position to be uncertain and hypothesize that they start at the closest TSS. The longest polycistronic transcript annotated by this study is the bicistronic UL37-36 RNA molecule spanning 13,041 nts.

*Complex transcripts* contain multiple genes of which at least one of them is encoded in the opposite strand. This study revealed a more intricate network of complex (cx)RNA molecules in BoHV-1 than in other herpesviruses^21,38,49^, for which the probable reason is technical: in this study we used high-coverage dRNA and dcDNA sequencing techniques. We identified a total of 76 complex transcripts. Twenty-one of these are represented by only one read and were detected by our screening of the longest isoforms. A putative transcript, the bICP22-AT3-SP2 spans almost the entire US region, starting from the TSS of bICP22 and terminating after the ATG of the US9 gene. Multigenic transcripts were already detected at 2 h p.i., and continued to be present throughout the entire course of infection (Supplementary figure S3). Complex isoforms increased their count 20-fold starting from 8 h p.i., indicating the increase of the read-through events at the later stage of infection.

### Intergenic peptides mapping to transcript isoforms

We used the proteogenomic coordinates of intergenic peptides detected by a previous tandem mass spectrometry experiment^8^ to analyze the isoforms’ coding potential. For 95 isoforms the peptides mapped to the 5′-UTR, 16 in-frame with an ORF with canonical start and end sites. Fifty-six of these transcripts were long 5′-UTR isoforms of 28 complex transcripts. Peptides also mapped to the 3′-UTR of 31 isoforms, 10 of them being in-frame with a canonical ATG and stop codon. Detection of these peptides suggests translational activity on the 5′- and 3′-UTR region of some viral genes.

Eighty-one putative protein-coding transcripts are also overlapped by a peptide, however these are out-of-frame with respect to the main genomic ORF or its truncated version, and no canonical ATG or stop codon is present for these peptides. Jefferson and colleagues suggest, that the apparent lack of a canonical ORFs might originate from frame shifting events caused by splicing, however our results did not confirm this hypothesis. Peptides originating from non-canonical ORFs overlapped 16 of the non-coding isoforms including seven 5′-, three 3′-truncated and 6 antisense transcripts. These however do not support the *in-silico* coding potential estimation, since CPAT assesses only canonical ORFs. For a comprehensive list of intergenic peptide locations see Supplementary table S3.

### A complex meshwork of transcriptional overlaps

This study revealed an extreme complexity of transcriptional overlaps, which is generated by frequent transcriptional readthroughs and head-to head overlaps produced by divergent genes. Transcriptional readthrough is already a well-known phenomenon between co-terminal tandem gene clusters. Here, we demonstrated that even more distal genes with their own transcription termination are able to carry out occasional transcriptional readthroughs. With a few exceptions (UL30-31, LR-ORF1-bICP0, LR-ORF2-bICP0), canonical transcripts of convergent gene pairs terminate without overlapping each other. Furthermore, we show that every convergent gene produces sporadic readthroughs with a mean overlap size of 153.5 nt (σ = 705.6). The most extensive overlap was detected between two putative transcripts UL18-17-16-15-14-13-12-11-C1 and UL15-L4-SP1 with a size of 9498 nts.

## Discussion

The past decade has witnessed tremendous advances in long-read sequencing. Besides the Pacific Biosciences and Oxford Nanopore Technologies platforms, Loop Genomics has also developed an LRS technique based on single molecules synthetic long-read sequencing. High-throughput LRS techniques are able to read full-length RNA molecules, which led to the discovery that the transcriptomes of examined organisms are much more intricate than previously thought. LRS-based studies have demonstrated that herpesviruses exhibit astoundingly complex transcription profiles^21,38,49^. Here we report the assembly and annotation of the BoHV-1 dynamic transcriptome. Our analysis identified a large number of novel transcripts and transcript isoforms. Our results demonstrate that practically every BoHV-1 gene produces transcripts with one or more 5′-truncated in-frame ORFs. It is unknown whether these ORFs are translated, and if so, what their functional significance could be. Several novel splice sites were also identified in this work. This result suggests that splicing in alphaherpesviruses may be more common than previously thought. However, in most cases, splice isoforms are expressed in low abundance relative to the common variant. It can be speculated whether the relative abundance of the splice isoforms is dissimilar in different cell types. We detected a huge variety of bICP22 splice isoforms and found that splicing occurred in the 5′-UTR of the transcript that overlaps two asRNAs. It is possible that these asRNAs contribute to the alternative splicing of bICP22, as suggested in other systems^50,51^. Novel introns in the UL40 and UL15 genes give rise to a change in amino acid composition. The increase of UL40-SP1 following viral replication might play a role in the virus’ transition into post-replication-phase. Replication-associated RNAs have been shown to regulate replication by altering primer synthesis^52^ or mediate the recruitment of the Origin Recognition Complex^53^. We discovered six replication-associated RNAs (ORIS-RNA1, ORIS-RNA2 and four 5′ UTR isoforms of bICP22), which overlaps the OriS of the virus. ORIS-RNA1 and ORIS-RNA2 are non-coding raRNAs and have no homologous counterparts in closely related viruses. This finding is consistent with observations in closely related herpesviruses.

In HSV-1, a long TSS isoform of ICP4 transcript overlaps the OriS, whereas in PRV the non-coding PTO^32^ and the long TSS variant of the US1 transcript overlap the OriS. It appears to be that raRNAs underwent very rapid evolution, and every species has developed unique solutions for raRNA production. Additionally, two miRNAs have been reported to overlap both raRNAs in an antisense orientation^14^, which may play a role in the regulation of raRNAs. Our study demonstrated the existence of very long polycistronic transcripts, and also that genes thought to be present in only bi- or polycistronic RNAs, are also expressed as monocistronic transcripts. A great diversity of transcription initiation is also described in this report. The same ORFs are expressed in a large number of TSS isoforms, with many starting in adjacent genes, and some in more distal genes. A large fraction of this variation is represented by low-abundance transcripts. 5′-UTRs were shown to modulate translation by forming specific structures^54^, or through upstream AUGs or uORFs^48,55,56^. Two-thirds of the overlaps between previously detected peptides and the transcript isoforms mapped to the 5′-UTR, suggesting the translation of potential uORFs. The function of the great extent of TSS variation in BoHV-1 is currently unknown; however, it may lead to post-transcriptional modifications, or differential translation. The prototypic organization of the herpesvirus transcriptome is characterized by the 3′-co-termination of the transcripts produced by tandem genes. Our findings suggest TES variation being substantially lower than TSS diversity. However, transcriptional readthrough is common not only in the parallel, but also in convergent genes. The 3′-UTRs may contain cis-acting elements determining the fate of RNAs after transcription^57^. The 3′-UTR of some isoforms also showed translational activity. A recent paper by Wu and colleagues proposed that downstream ORFs have a translation-enhancing function^58^, which needs further confirmation in viruses.

An extremely complex meshwork of transcriptional overlaps was also described. Overlaps are produced by transcriptional read-throughs between tandem and convergent genes or by the mutual utilization of certain genomic loci by divergent genes. Multiple transcriptional read-throughs can result in the generation of complex transcripts. Convergent and divergent transcriptional overlaps produce antisense RNA segments on transcripts. These overlaps may give rise to double-stranded RNAs (dsRNAs), which are targeted by the dsRNA-activated protein kinase R^59^ or type I interferon system^60^ of the host, thereby effectively reducing viral translation. In a recent publication Dauber and co-workers reported the mechanism of how HSV-1 virion host shut-off (VHS) protein limits the accumulation of viral dsRNAs^61^. In light of these findings, it is possible that the frequency of RNA polymerase II readthroughs are much higher than expected from the basis of ratio of read-thorough transcripts.

The high-coverage of sequencing reads allowed us to carry out kinetic characterization of viral transcripts. We used dcDNA data because it produces longer reads than both amplification-based and native RNA sequencing techniques. At the same time, dcDNA-Seq lacks the potential amplification biases causing improper quantitation^62,63^. LRS allows the distinction between parallel-overlapping RNA molecules and transcript isoforms, therefore we can not only kinetically characterize the genes, but also transcript isoforms.

These analyses and data provide valuable resources for future functional studies.

## Methods

### Cells and viruses

Cultured cells were infected with the Cooper isolate (GenBank Accession # JX898220.1) of Bovine Herpesvirus 1.1. Viruses were maintained and propagated in two different laboratories (one in Mississippi State University, Starkville, Mississippi, USA, and the other one in Institute for Veterinary Medical Research, Budapest, Hungary).

#### BoHV-1 from Mississippi (BoHV-1/MS)

Madin Darby Bovine Kidney (MDBK) cells were incubated at 37 °C in a humidified incubator with 5% CO_2_, and were cultured with Dulbecco’s modified Eagle’s medium (DMEM) supplemented with 5% (v/v) fetal bovine serum, 100 U/mL penicillin, and 100 µg/mL streptomycin. Cells were either mock-infected or infected with Cooper isolate (GenBank Accession # JX898220.1) of Bovine Herpesvirus 1.1 (BoHV-1). Due to the short duration of the infection, we used a multiplicity of infection (MOI) of 5 plaque-forming units (PFU)/cell to minimize the number of cells that remain uninfected in the dish. Cells were incubated at 4 °C for 1 h for synchronization of infection, and then placed in a 5% CO_2_ incubator at 37 °C. Infected cells were collected at 1, 2, 4, 6, 8, and 12 h post infection (HPI). Each time-point and mock infection consisted of three replicates (n = 3). Cells were washed with phosphate buffered saline (PBS), scraped from the culture plate and centrifuged at 300 RPM for 5 min at 4 °C.

#### BoHV-1 from Hungary (BoHV-1/HU)

The Cooper strain of BoHV-1 was propagated in ovine kidney (OK) cells (ATCC CRL-6551). OK cells were grown to complete confluency in DMEM (Life Technologies) culture medium containing 10% new-borne calf serum (NCS) and antibiotics. Cells were infected with virus stocks at MOI = 1, and grown in DMEM without NCS and incubated at 37 °C in a humidified 5% CO_2_-in-air atmosphere incubator. Cells were washed with phosphate buffered saline (PBS), scraped from the culture plate and centrifuged at 300 RPM for 5 min at 4 °C. Virus stocks were stored at − 80 °C.

### RNA isolation

BoHV-1/MS and BoHV-1/HU: RNA from infected and uninfected cells (MDBK cells for BoHV-1/MS or OA cells for BoHV-1/Hu) was extracted using the NucleoSpin RNA kit (Machery-Nagel, Bethlehem, PA, USA), with the lysis step augmented by the addition of proteinase K (final concentration 0.37 mg/mL).

### Poly(A) RNA selection and rRNA depletion

For the analysis of the polyadenylated RNAs, this RNA fraction was enriched using Oligotex mRNA Mini Kit (Qiagen). To obtain potential non-polyadenylated transcripts, rRNA depletion was performed using Ribo-Zero Magnetic Kit H/M/R (Epicentre/Illumina).

### ONT: direct RNA sequencing

The BoHV-1/HU samples were pooled in equimolar ratios and used for the preparation of (dRNA) libraries using ONT Direct RNA Sequencing Kit (SQK-RNA001). The cDNA was synthetized using SuperScript IV Reverse Transcriptase (Thermo Fisher Scientific) and an RT adapter containing an overhang of 10 Ts, and 500 ng poly(A) + RNA. RT adapters (supplied in the kit) were ligated to the RNA strand using T4 DNA ligase (New England Biolabs).

### ONT: direct cDNA sequencing

Non-amplified cDNA libraries were prepared from the mock and six BoHV-1/MS p.i samples in three replicates using the ONT’s Direct cDNA Sequencing Kit (SQK-DCS109) according to the manufacturer’s instructions. Briefly, first strand synthesis was performed using Maxima H Minus Reverse Transcriptase (Thermo Fisher Scientific) with SSP and VN primers (supplied in the kit) and 100 ng of poly(A) + RNA for each sample. This was followed by the elimination of potential RNA contamination using RNase Cocktail Enzyme Mix (Thermo Fisher Scientific), and second strand synthesis using LongAmp Taq Master Mix (New England Biolabs). Double stranded cDNA ends were repaired using NEBNext End repair /dA-tailing Module (New England Biolabs) and consecutive sequencing adapter ligation employing the NEB Blunt /TA Ligase Master Mix (New England Biolabs).

Libraries were barcoded using Native Barcoding (12) Kit (ONT) according to the manufacturer’s instructions.

### ONT: amplified cDNA sequencing libraries

Amplified cDNA libraries were produced for both the BoHV-1/MS and BoHV-1/HU samples using ONT Ligation Sequencing Kit 1D (SQK-LSK109), with the BoHV-1/MS samples being previously pooled in equimolar ratios. Briefly:

#### RT with oligo(dT) primers

50 ng of poly(A) + RNA was reverse transcribed using SuperScript IV Reverse Transcriptase and oligo(dT) primers (supplied in the kit). The cDNA samples were subjected to PCR using KAPA HiFi DNA Polymerase (Kapa Biosystems) and Ligation Sequencing Kit Primer Mix. End repair and sequencing adapter ligation were carried out as described for the dcDNA-Seq library preparation.

#### RT with random primers

50 ng of ribodepleted RNA was reverse transcribed using SuperScript IV Reverse Transcriptase and custom-made primers composed of a random hexamer sequence and one complementary to Ligation Sequencing Kit Primer (supplied in the kit). PCR, end repair and sequencing adapter ligation were identical to the oligo(dT) primed RT library. The resulting four libraries were barcoded using the 1D PCR Barcoding (96) Kit (Oxford Nanopore Technologies) following the manufacturer’s instructions.

### LoopSeq single-molecule synthetic long-read sequencing

LoopSeq libraries were prepared from multiplexed 2 h and 12 h post infection samples in three replicates using LoopSeqTM Transcriptome 3 × 8-plex Kit. Phasing mRNA protocol was performed according to the manufacturer’s instructions.

### Purification and concentration measurement of the libraries

Libraries were purified after each enzymatic steps using Agencourt AMPure XP magnetic beads or for dRNA-Seq, the RNAClean XP beads (both from Beckman Coulter). Qubit RNA BR and HS Assay Kits and Qubit DNA HS Assay Kit (Thermo Fisher Scientific) were used to measure the total RNA, poly(A) + RNA, and cDNA concentrations, respectively.

### Sequencers

Sequencing of the ONT dRNA, dcDNA and amplified cDNA libraries was performed on R9.4.1 SpotON Flow Cells (ONT). To avoid barcode cross-talk from later time points, mock-infected, 1 h and 2 h p.i. samples were sequenced separately from other samples. The LoopSeq library was sequenced on an v2 300 flow cell on the Illumina MiSeq system.

### Pre-processing and data analysis

The MinION data was base called using Guppy base caller v. 3.4.1. with—qscore_filtering. Reads with a Q-score greater than 7 were mapped to the circularized viral genome (NCBI nucleotide accession: JX898220.1) with the Minimap2 software (Li, 2018). The same reads were also mapped to the host genomes, as follows: the BoHV-1/MS sample was mapped to the genome assembly of Bos taurus (GCF_002263795.1) while the BoHV-1/HU sample to the genome of Ovis aries (GCA_002742125.1), both using the Minimap2 aligner.

Synthetic long-read data was base called on the MiSeq device using the Real-Time Analysis software with default settings. Base called reads were then processed by the Loop Genomics Pipeline Software with default settings. Synthetic long reads were mapped to the BoHV-1 and Bos taurus genomes using Minimap2.

#### Structural analysis

For transcript isoform detection and annotation, mapped reads were analyzed with the LoRTIA software suite v.0.9.9, using the following steps: (1) To eliminate reads resulting from partial RT or PCR and artefacts resulting from false priming non-trimmed read ends were searched for the sequencing adapters for the TSS or the presence of a homopolymer A sequence for the TESs. The first nucleotide not aligning with the adapter was denoted as possible TSS while the last nucleotide not aligning with the homopolymer A was detonated as putative TES. Any other read start/end position was discarded. (2) Putative TSSs and TESs were tested against the Poison distribution to eliminate random start and end positions caused by RNA degradation. The significance is corrected with the Bonferroni method. Features failing to pass qualifying as local maxima, or being present in less than 2 reads or in less than 1‰ of the coverage are eliminated from the analysis. (3) Putative introns are annotated if they have one of the three most commune splice consensus sequences (*GT/AG, GC/AG, AT/AC*) and they are more abundant than 1‰ compared to the local coverage. Introns with splice junctions flanked by tandem repeats are removed from the analysis as these can be template switching artefacts.

The resulting putative TSSs and TESs were considered as existing if they were detected in at least three independent samples. Deleted segments were accepted as introns if they were present in both dRNA-Seq and one of the cDNA-Seq datasets and if they were shorter than 10 kbps. We set a relatively low abundance for acceptance because it may vary in different cell types. The accepted TSSs, TESs and introns were then assembled into putative transcripts using the Transcript_Annotator software of the LoRTIA toolkit. Very long unique or low-abundance reads which could not be detected using LoRTIA were annotated manually. These reads were also accepted as putative transcript isoforms if they were longer than any other overlapping RNA molecule. In some cases, the exact TSSs were not annotated. Finally, a read was considered as transcript if it was present in at least three separate samples. Transcript annotation was followed by isoform categorization according to the following principles: the most abundant transcript containing a single ORF was termed canonical monocistronic transcript, whereas isoforms with longer or shorter 5′-UTRs or 3′-UTRs regions than the canonical transcripts were termed TSS or TES isoforms (variants), respectively. Similarly, transcripts with alternative splicing were named splice isoforms. Transcripts with 5′-truncated in-frame ORF were termed as putative mRNAs. Transcripts with multiple non-overlapping ORFs were designated polycistronic, whereas those with ORFs in different orientation were called complex transcripts. Transcripts with no ORFs or ORFs shorter than 30 nts were named non-coding, except if occurred in front of a canonical ORF in a transcript (These small ORFs were termed uORFs).

#### Analysis of viral gene expression

For the characterization of viral gene expression kinetics, we used dcDNA-seq datasets annotated by LoRTIA. First, we filtered out reads that were confirmed by less than 4 other reads in the dcDNA-seq datasets, then we normalized read counts using a modified version of the Median Ration Normalization of the DESeq2 software suite (Wu et al., 2019). The average normalized read counts of the biological replicates was then considered as the read count of a given isoform.

### Coding potential analysis

The assessment of the coding potential of the long non-coding RNAs was performed using the Coding Potential Assessment Tool (CPAT)^40^ with the standard vertebrate codon table. The non-redundant NCBI protein database was also searched for ORF homologies. To infer translational information for the vast variety of structural isoforms detected we compared our results to a previous proteogenomic analysis of the virus. The coordinates of intergenic peptides with and without a viable ORF detected by Jefferson et al.^8^ were overlaid with the resulting isoforms, and their location within the transcript was annotated.

A schematic representation of the workflow can be found in Supplementary Figure S4.

### Ethics declaration

Neither human nor animal experiments were applied in this study.

## Supplementary information

Supplementary Information 1.

Supplementary Information 2.

Supplementary Information 3.

Supplementary Information 4.

Supplementary Information 5.

## Data Availability

The LoRTIA software suite is available on GitHub: https://github.com/zsolt-balazs/LoRTIA.Our in-house scripts used to generate the descriptive statistics of reads and transcripts, to analyze promoters and to detect transcript isoforms are available on GitHub: https://github.com/moldovannorbert/seqtools. The sequencing datasets generated during this study are available at the European Nucleotide Archive’s SRA database under the accession PRJEB33511 (https://www.ebi.ac.uk/ena/browser/view/PRJEB33511). The transcript annotations are available in the Supplementary Dataset 1.
