# Supplementary material for: Time-course profiling of bovine alphaherpesvirus 1.1 transcriptome using multiplatform sequencing
Source: Sci Rep. 2020 Nov 24;10:20496. doi: 10.1038/s41598-020-77520-1 (PMC7686369; doi:10.1038/s41598-020-77520-1)
Supplement: Supplementary file 2 — Supplementary Information 2. [file 41598_2020_77520_MOESM2_ESM.docx]

**Time-course profiling of bovine alphaherpesvirus 1 transcriptome using multiplatform sequencing**

Norbert Moldován, Gábor Torma, Gábor Gulyás, Ákos Hornyák, Zoltán Zádori, Victoria A. Jefferson, Zsolt Csabai, Miklós Boldogkői, Dóra Tombácz, Florencia Meyer, Zsolt Boldogkői


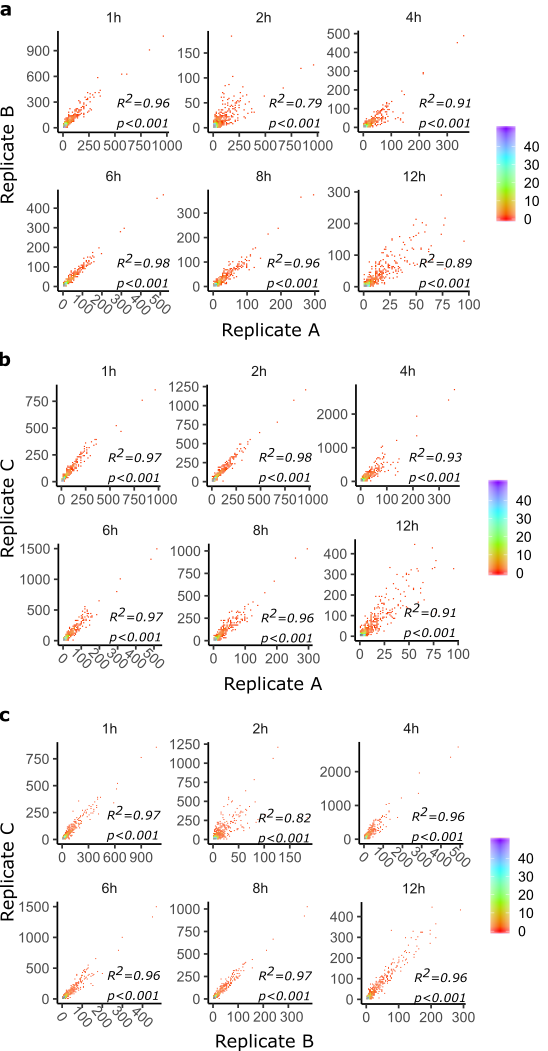


**Supplementary Figure S1. Reproducibility of the results.** Pearson’s correlation of the read counts for **a.** Replicate A and B, **b.** Replicate A and C and **c.** Replicate B and C. Data density is show in colors on the right of the scatter plots, where blue and purple represents mora abundant data points. A high correlation was detected among the read counts of the replicates.


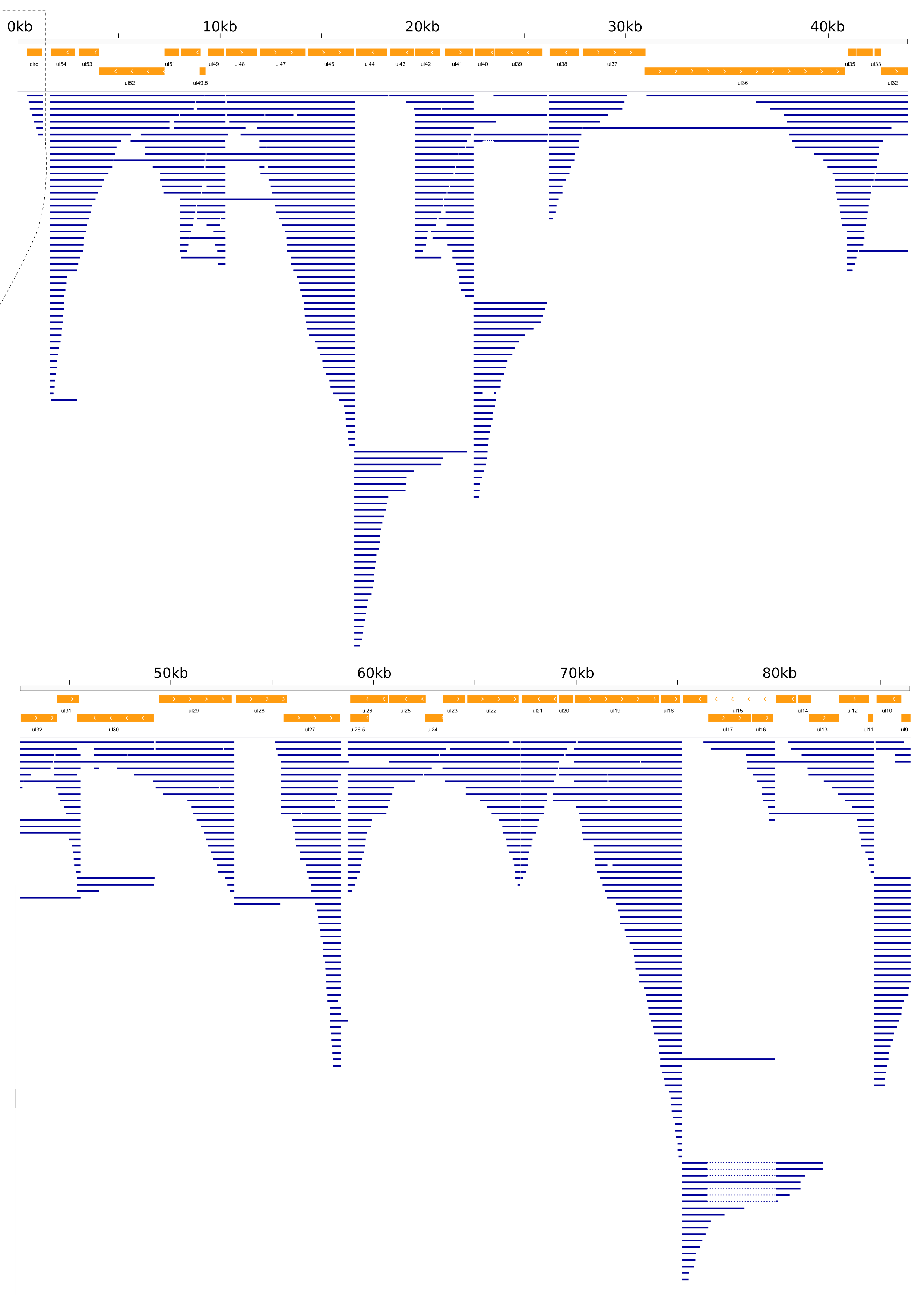


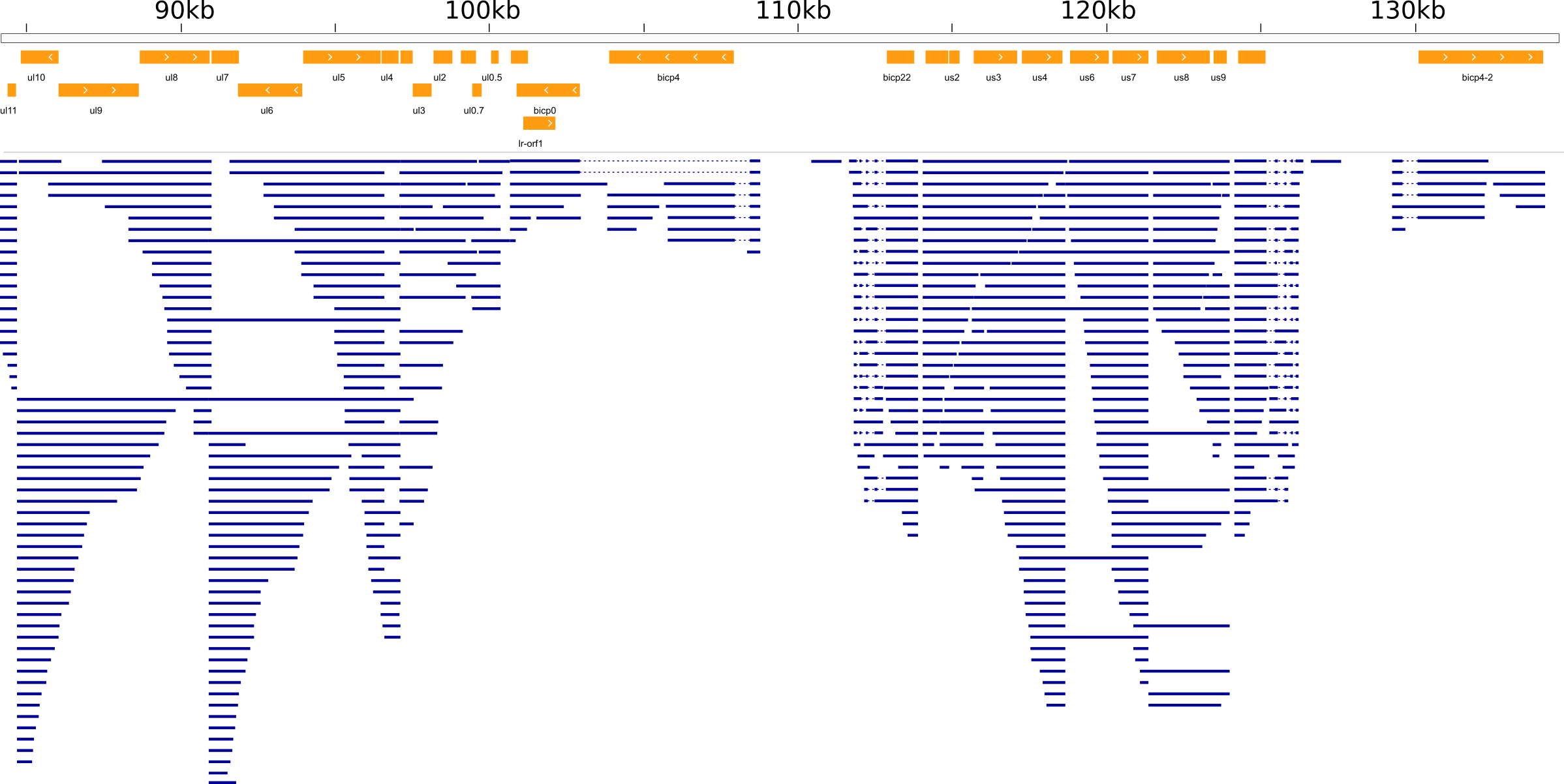

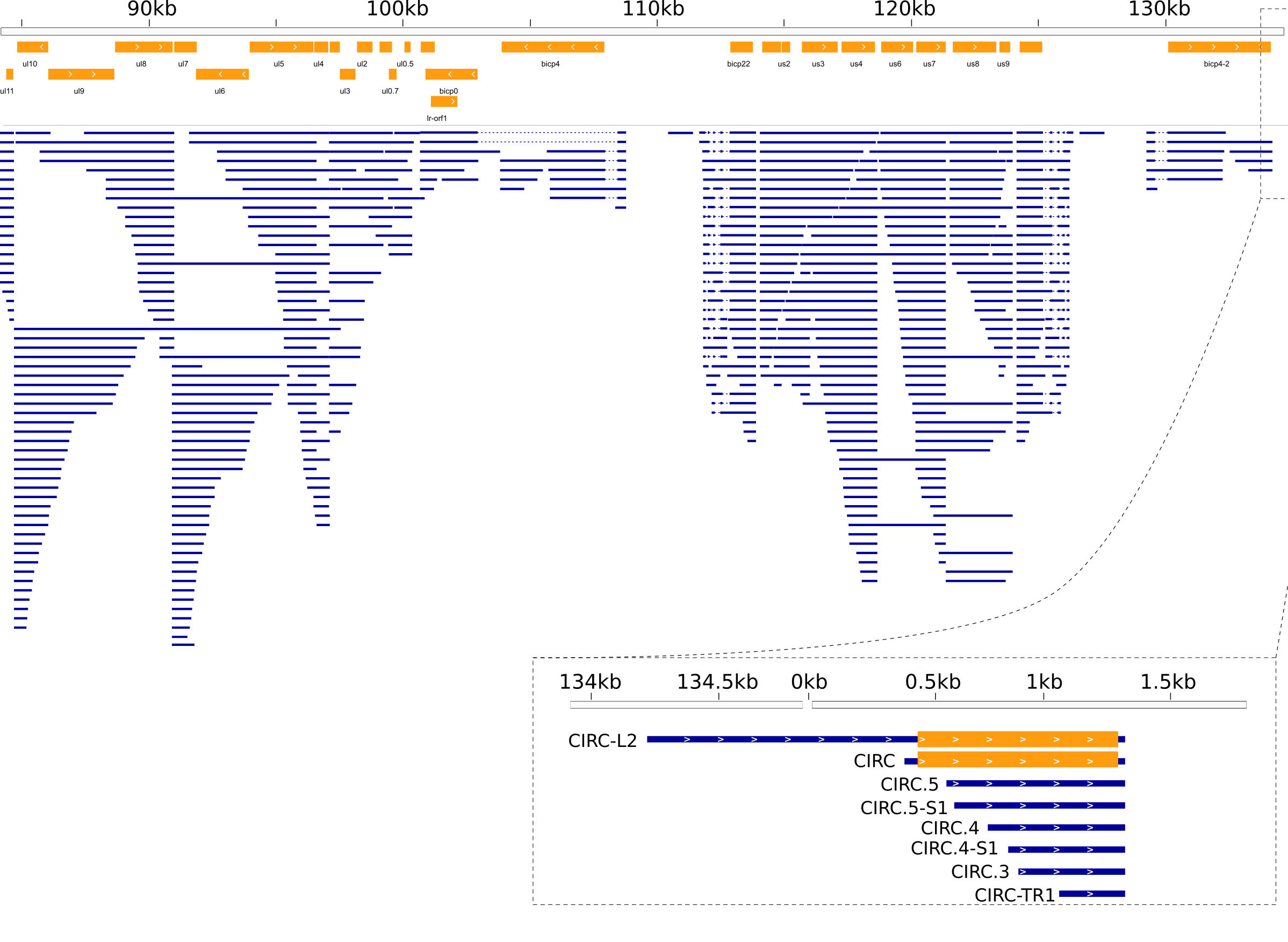
**Supplementary Figure S2. The transcriptome of BoHV-1.** Orange rectangles represent ORF-s while blue lines the transcripts present in at least 3 samples. Dotted lines between blue lines represent introns. Transcripts are aligned to the BoHV-1 genome with accession number: JX898220.1. The genomic junction is enlarged, showing the overlapping CIRC-L2 transcript.


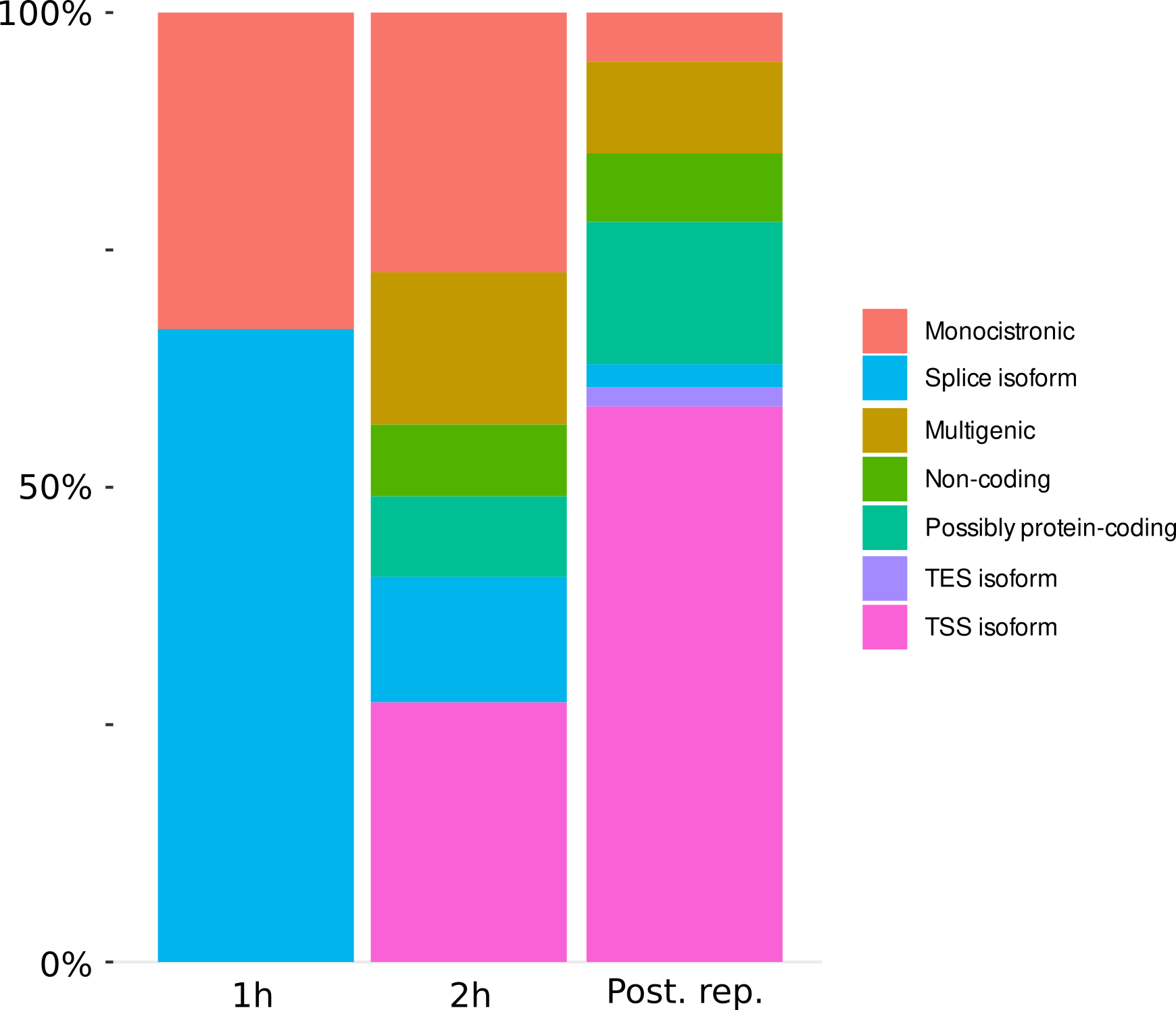


**Supplementary Figure S3. The proportion of viral transcript isoforms in phases of the infection.** Isoform categories were merged for better visualization as follows: Moncistronic: monocistronic transcripts; Splice isoforms: spliced transcripts and non-spliced isoforms of transcripts previously known to be spliced; Multigenic: bi-, tri-, polycistronic and complex transcripts; Non-coding: 5’- and 3’-truncated transcripts without an ORF and antisense and non-coding transcripts; Possibly protein-coding: 5’-truncated transcripts with truncated ORFs; TES isoforms: transcripts with alternative terminations; TSS isoforms: transcripts with shorter and longer 5’ UTRs.


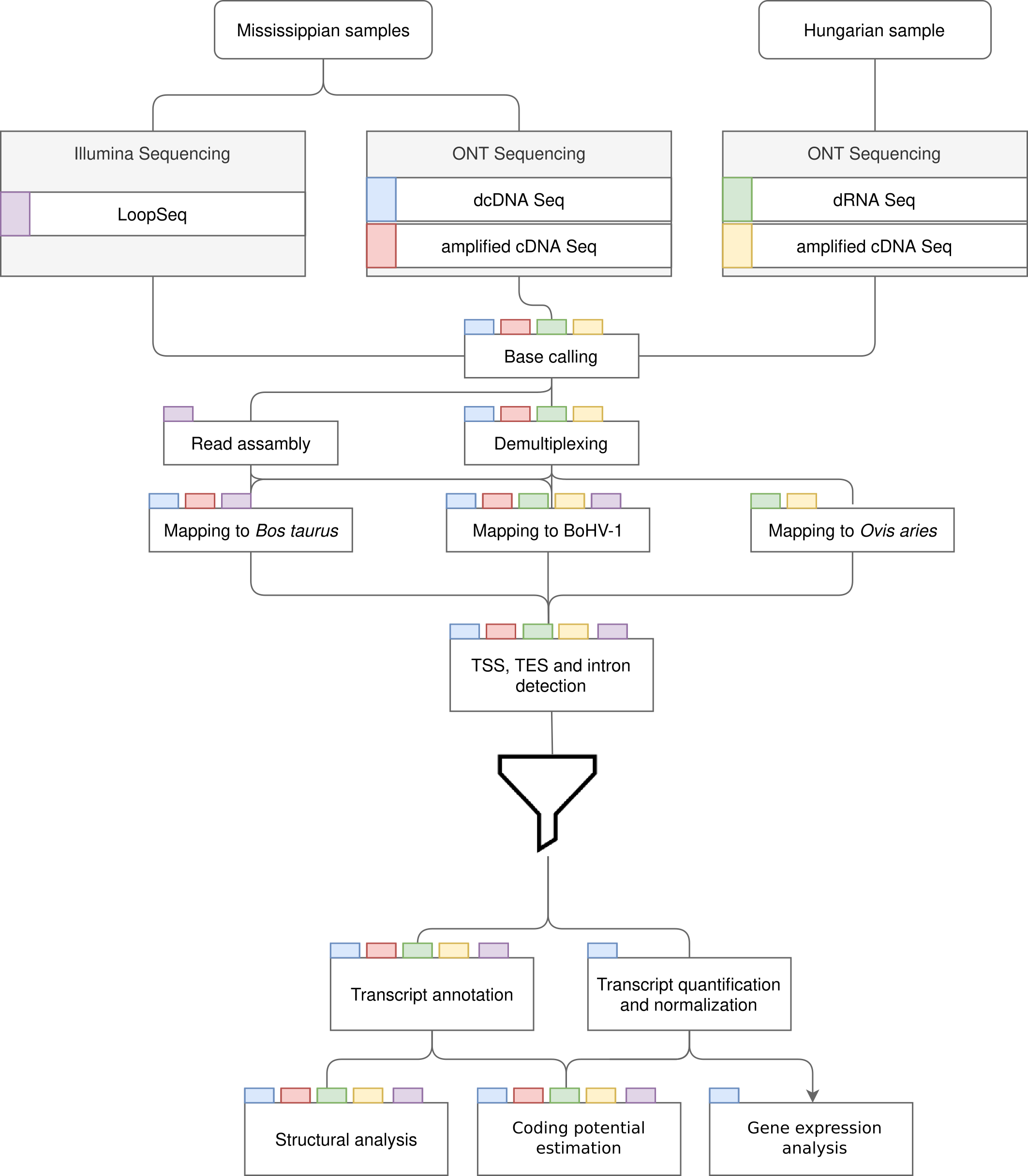


**Supplementary Figure S4. Schematic representation of the workflow.** Colored rectangles represent the sequencing libraries. Steps of data analysis are shown in rectangles, with the libraries undergoing the given step indicated by the tab color. The funnel symbol represents TSS, TES and intron filtering.

**SUPPLEMENTARY TABLE LEGENDS**

**Supplementary Table S1. TSSs, TESs and introns of BoHV-1 detected by LoRTIA.** Nucleotide positions are given based on the genome with accession number: JX898220.1.

**Supplementary Table S2. Promoters and polyadenylation signals of viral transcripts.**

**Supplementary Table S3. Putative peptides with canoncal and non-canonical ORFs located on transcript isoforms and coding potential assessment.** Genomic locations are according to the BoHV-1 genome with accession number JX898220.1. Sheet ‘Non-canonical ORFs’ shows the location of detected peptides, sheet ‘Canonical ORFs’ shows the ORFs relative to transcript isoforms, while sheet ‘CPAT results’ shows the resulst of the coding potential assessment.

**Supplementary Data S1. The transcript annotations of the BoHV-1 detected by LoRTIA.** Nucleotide positions are given based on the genome with accession number: JX898220.1.
